# Gender Variance Among Youth with Autism Spectrum Disorders: A Retrospective Chart Review

**DOI:** 10.1089/trgh.2015.0007

**Published:** 2016-02-01

**Authors:** Aron Janssen, Howard Huang, Christina Duncan

**Affiliations:** ^1^ NYU Child Study Center, New York, New York.; ^2^ New York University, New York, New York.

**Keywords:** child and adolescent development, gender dysphoria, mental health

## Abstract

**Purpose:** Increasing clinical evidence suggests an overrepresentation of gender variance (GV) among patients with autism spectrum disorders (ASDs). This retrospective chart review aims to contribute to the existing literature on co-occurring ASD and gender dysphoria (GD). We compare the rate of parent-reported GV in patients with an ASD diagnosis to that of parent-reported GV in a normative nonreferred data set.

**Methods:** Child Behavior Checklist (CBCL) charts were collected from 492 children and adolescents (409 natal males and 83 natal females) aged 6–18 years who have received a diagnosis of ASD at the New York University Child Study Center. Parent-reported GV was determined through endorsement of CBCL sex item 110, which assesses the presence of gender-related issues. We calculated the odds ratio of endorsement of item 110 between our ASD sample and the CBCL sample data.

**Results:** The subjects diagnosed with ASD were 7.76 times more likely to report GV than the CBCL sample. This finding was statistically significant. About 5.1% of the patients in the ASD group and 0.7% of the CBCL nonreferred group endorsed sex item 110. 5.1% of natal males and 4.8% of natal females endorsed sex item 110. Neither gender nor age influenced the rate of endorsement.

**Conclusion:** This finding supports the growing research suggesting a heightened co-occurrence rate of ASD and GD. Focus should be placed upon improving our understanding of the nature of this co-occurrence and on gender identity development within the atypical development of ASD.

## Introduction

Autism spectrum disorders (ASDs) are a set of neurodevelopmental disorders that are characterized by impairments in social communication skills and repetitive and restricted interests and behaviors.^1^ There is increasing evidence that suggests an association between gender dysphoria (GD), gender variance (GV), and ASDs.^2–4^

GD is a DSM-5 diagnosis characterized by distress caused by a persistent incongruence between one's natal sex and expressed gender,^1^ and GV is a broader term that describes any numbers of variability between assigned sex and experienced/expressed gender. An increasing number of researchers and practitioners have noticed a high rate of co-occurrence between GD and/or GV and ASDs. Many case studies have suggested an overrepresentation of GD among clients with ASDs and vice versa.^5–8^ De Vries et al.^4^ systematically measured this overrepresentation in a large-scale Dutch-based study. Among clients who presented for evaluation at a gender clinic in Amsterdam, a prevalence rate of 7.8% was reported for ASDs. This is much higher than the prevalence rate expected for the general population in Northern Europe, which ranges from 0.3% to 1.16%.^9^ Another study based in North America examined this association in the opposite direction.^3^ It was found that 5.4% of the subjects diagnosed with ASD answered positively for sex item 110 (“wish to be opposite sex”) on the Child Behavior Checklist (CBCL).^3,10^ This is significantly higher than the rate of positive response expected of the general population, 0.7%.^10^

Despite these convincing results and their obvious clinical implications, literature on the co-occurrence of ASD and GD remains inchoate. The present study is a retrospective chart review that aims to contribute to this topic by exploring the following hypotheses in a child and adolescent mental health clinic based in New York City:

1. In congruence with previous large-scale findings,^3,4^ we expect a heightened rate of endorsement of sex item 110 on the CBCL in our local ASD sample compared to the nonreferred normative CBCL standardization data.^10^
2. Previous studies have suggested similar rates of gender-related issues between natal males and females diagnosed with co-occurring ASD.^3,4^ We expect to find similar rates of endorsement of sex item 110 between natal males and natal females within the local ASD sample.
3. Previous research has consistently shown a high rate of desistance in transgender identity among children as they grow into adolescence.^11,12^ We hypothesize that this trend of desistance may also be present among the local ASD samples, presented by a negative correlation between age and rate of endorsement of sex item 110.

## Methods

All study activities were approved by the Institutional Review Board at the NYU Langone Medical Center, and patient data were protected by strictly following the approved methods and use of deidentified data. All data were manually entered into IBM SPSS Statistics, version 23. Descriptive statistics (frequencies, means, and standard deviations [SD]) were calculated. Significance tests (chi-square test, Fisher's exact test, and independent *t*-tests), odds ratios, and logistic regression models were calculated on R and the package “psych.”^13^

The present study is a retrospective chart review. CBCLs were collected from all patients who were between ages 6 and 18 years and had received formal diagnoses of ASDs (i.e., ASD, Asperger's syndrome, pervasive developmental disorders, and all associated not otherwise specified [NOS] diagnoses) between January 2011 and January 2015 at the New York University (NYU) Child Study Center in midtown Manhattan, New York City. Using this method, CBCL charts were pulled from 492 subjects. The average age of the sample was 8.96 years (SD=2.704, age range: 6–18 years). Of these subjects, 409 (83.1%) were natal males and 83 (16.9%) were natal females. This is a fairly typical sex ratio of the ASD population, wherein males receive the diagnosis roughly four times more frequently than females.^9^

Normative data were obtained from the nonreferred standardization sample of the CBCL (*N*=1605).^10^ Participant characteristics of both normative control group and our local sample group are summarized in Table 1. Compared to the normative CBCL data, our sample was significantly younger, *t* (1022)=18.64, *p*<0.0001. This significant difference was addressed through logistic regression analysis and data manipulation. The natal sex ratio in our sample was significantly more male than that in the normative CBCL data (χ^2^=142.34, *p*<0.01). Data on race, ethnicity, and socioeconomic status were not available for comparison between the normative data and local sample.

**Table 1.** **Age and Biological Sex of Participants**

	Age, years			Biological sex, *N* (%)	
	*M*	SD	Range	Female	Male
CBCL normative sample (*N*=1605)	11.74	3.44	6–18	754 (47%)	851 (53%)
ASD (*N*=492)	8.96	2.70	3–17	83 (17%)	409 (83%)

ASD, autism spectrum disorder; CBCL, Child Behavior Checklist; SD, standard deviation.

CBCL charts are collected from all patients upon initial evaluation as a part of standard care at the NYU Child Study Center. CBCL is an extensive checklist for both parent and self-report in assessing internalizing and externalizing behaviors, as well as a number of other specific behavioral problems.^10^ In the present study, all CBCL charts had been filled out by the patients' parents.

The CBCL is considered a “gold standard” for child and adolescent clinical evaluations. The present study looked particularly at sex item 110 on the CBCL, “wish to be opposite sex.” Endorsement of sex item 110 with the responses “sometimes true” or “very true” has been found to have strong correlations with clinical diagnoses of gender-related issues^14^ and has been used in previous work investigating the co-occurrence between ASDs and GV.^3^ As with all other questions on the questionnaire, three response choices are given for sex item 110: never true, somewhat or sometimes true, and very true or often true. For the purpose of analyses, two groups were formed: those who answered the item negatively (never true) and those who answered positively (somewhat or sometimes true and very true or often true).

It is worth mentioning, however, that endorsement of sex item 110 does not equate with a DSM-5 diagnosis of GD. In fact, it has been found that among a gender variant Dutch sample, Gender Identity Disorder (GID)-NOS or transvestic fetishism appeared to occur more frequently among the gender referrals with ASD than among those without ASD. However, a majority of the patients in the study with ASD still met criteria for GID and were found to be appropriate for medical/surgical intervention.^4^ Sex item 110 only assesses the presence of possible gender-related issues and GV and cannot provide further insight as to the particular clinical presentation or formal diagnoses of the child.

## Results

### Hypothesis 1

**FIG. 1.**
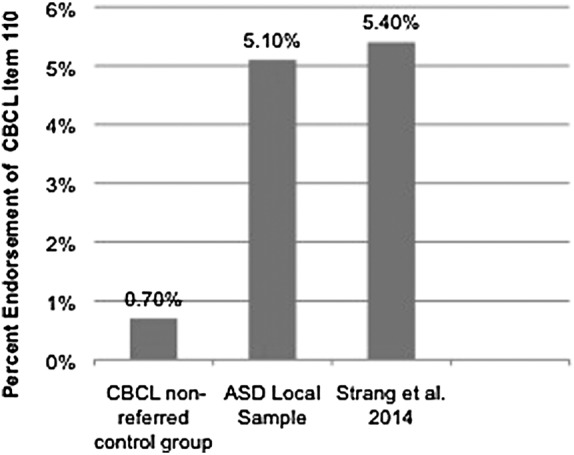
Percent endorsement of Child Behavior Checklist (CBCL) item 110 in the nonreferred control group, the autism spectrum disorder (ASD) local sample, and Strang et al.^3^

About 5.1% of the local ASD sample endorsed sex item 110. Of the ASD sample in Strang et al.,^3^ 5.4% endorsed sex item 110, as did 0.7% of the nonreferred sample from CBCL standardization sample.^10^ Percentages of endorsement by parents of item 110 among samples from our local clinic, Strang et al.,^3^ and CBCL nonreferred data are shown in Figure 1. Odds ratio was calculated between our ASD group and the nonreferred sample from CBCL nonreferred normative data. Compared to the CBCL group, local subjects diagnosed with ASDs were 7.76 times more likely to report GV, which was statistically significant. Table 2 summarizes the odds ratio (OR), 95% confidence intervals, and *p*-values of the present study and those of Strang et al.^3^

**Table 2.** **Odds Ratios for Endorsement of Sex Item 110 Between ASD Samples and the CBCL Nonreferred Control Group**

	CBCL nonreferred controls		
	Odds ratio	95% CI	*P*
Local ASD sample	7.76	3.79–15.88	0.001
Strang et al.^3^	7.59	3.05–18.87	0.001

CI, confidence interval.

In response to the significant age difference between the local ASD sample and CBCL normative data, we randomly deleted participants younger than 10 years until the average age of the manipulated data set matched that of the CBCL data. This procedure, along with OR calculations, was repeated three times. Calculated ORs were statistically significant for all three manipulated data sets and ranged from 8.25 to 9.22. It should be noted, however, that 325 participants had to be deleted each time, leaving the manipulated sample sizes to be 167. Nonetheless, logistic regression from hypothesis 3 revealed no significant age effect on the rate of endorsement. It is thus reasonable to infer that the rate of endorsement of sex item 110 was heightened across all age groups in our ASD sample.

### Hypothesis 2

Of the 492 subjects reviewed, 25 endorsed sex item 110 (5.1%). Of these 25 individuals, 21 were natal males (5.1% of all 409 natal males) and 4 were natal females (4.8% of all 83 natal females). A Fisher's exact test revealed no significant level of gender effect on the rate of endorsement (*p*=1).

### Hypothesis 3

To evaluate the potential effect of age on the rates of endorsing sex item 110, we modeled the indicator for endorsement as a function of age using logistic regression. There was no evidence that age is related to the endorsement of sex item 110 (*b*=0.056, se(*b*)=0.074, Wald's *t*=0.571, *p*=0.45).

## Discussion and Limitations

The present study joins a gradually growing body of literature on the co-occurrence of ASD and gender-related issues among child and adolescent clinical populations. In comparing a local sample of patients diagnosed with ASDs with nonreferred normative data, we find an elevated rate of GV. It is found that endorsement of sex item 110, “wish to be opposite sex,” is 7.76 times more likely among participants with an ASD diagnosis than in a nonreferred comparison group. The elevation in endorsement remains significant even after several adjustments for age differences between our local ASD sample and CBCL normative data. This finding is in congruence with those from several previous studies.^3,4,15,16^ In particular, our results are highly comparable to those of Strang et al.^3^ In that study, as in ours, the response options of “sometimes true” and “very true” were counted as affirmative responses to item 110. Our study was not designed to differentiate between these two groups, and further investigation into its significance is warranted. With one of the largest ASD sample sizes (*N*=429) to date, the present study provides strong evidence to the co-occurrence of ASD and GV—a topic that has heretofore been largely documented through single-case studies.

Also congruent with previous findings, no gender effect has been found that contributes to the endorsement of GV among the local ASD samples. The rate of endorsement of sex item 110 among natal male participants (5.1%) is virtually the same as that of natal female participants (4.8%). However, there have been several recent studies that suggest differences in hormonal and physiological,^17^ perinatal,^15^ and clinical presentations between male and female patients who receive a co-occurrent diagnosis of ASD.^18^ Although no difference in prevalence rate of GV has been found between male and female ASD clients, further research on the etiological and symptomatic presentations between the two groups is encouraged.

A logistic regression reveals no age effect on the rate of endorsement of sex item 110. In this sample, younger children were as likely to endorse this item as adolescents. This is in contrast to the high rate of desistance in studies of childhood GV.^11,12^ However, without follow-up CBCL data, we cannot make conclusions about the persistence or desistance of GV of individuals included in this data set.

Generally, children demonstrate the ability to discriminate faces and voices by sex by 9–11 months^19^ and reach gender constancy by 5 years of age in stepwise progressions.^20–22^ There is preliminary evidence that, at least for gender distinction, individuals with ASDs may experience persistent impairment in gender-related developments.^23–26^ Furthermore, a study by de Vries et al.^4^ suggests that for some people with ASD, transgender identities may deviate from those of neurotypical individuals. Individuals with ASDs were much more frequently diagnosed with GID-NOS and transvestic fetishism than the neurotypical control group.^4^ Although literature in gender identity development among individuals with ASDs is limited, there is at least some evidence that suggests uniqueness in gender-related concerns among individuals with ASDs. We hypothesize that such uniqueness may influence the presentation, development, and expression of GV among some individuals with ASDs.

These findings have obvious conceptual and clinical implications. These findings contribute to the literature that suggests increased rates of GV among individuals with ASDs. Given this increased prevalence, practitioners working with youth with ASDs should screen for gender identity concerns. However, one must be careful not to infer a direct relationship with an affirmative response on the CBCL and a DSM-5 diagnosis of GD, particularly given the increased prevalence of transvestic fetishism and GID-NOS among individuals with ASDs.^4^ Gender development is a complex process involving biological, psychological, and cognitive elements that are influenced by social norms. As such, individuals with ASDs may present with variance in their gender identity and expression that may defy easy categorization. Clinical practice must take into account the social, cognitive, and developmental trajectories of patients with ASDs to personalize treatment plans and ensure that individuals with ASDs are able to maintain their autonomy and have equal access to gender-related care.

There are several limitations to the present study. First, the present study recruited clinical samples only, and future research is encouraged to explore the co-occurrence between ASD and GV in nonreferred populations.

Second, due to the retrospective nature of the present study, data collection was limited to preexisting measurements that provided only categorical data. Not all the desired demographic and clinical information was available in the data set. While the Autism Diagnostic Observation Schedule is most frequently used at the NYU Child Study Center to diagnose ASDs, the type of evaluation used to confirm the diagnosis of an ASD was not available in this data set. The atypicality of the presentation of GV among individuals with ASDs warrants more encompassing measurement. Although sex item 110 on the CBCL has been shown to accurately assess GV, more comprehensive measurements should be used in the future. Similar limitations are true for ASD-related measurements as well: Standardized measures of intellectual ability or ASD severity were unavailable for the present study. The use of standardized, comprehensive, and dimensional instruments for both autistic traits and GV is recommended for future research.

Last, the present study did not explore the potential etiological factors behind the co-occurrence of ASDs and GV. Future research is encouraged to collect data on a wide array of factors—in particular, birth weight,^15^ social awareness, and severity of ASD symptoms^3,15^ have been suggested as major factors behind this phenomenon and deserve further investigation.

## Conclusions

The present study is a large-scale retrospective prevalence study that confirms a heightened level of co-occurrence of ASDs and GV. It joins a growing body of literature that consistently suggests such co-occurrence. Longitudinal research is recommended to further our understanding of this phenomenon, especially in regard to gender identity development within the atypical developmental time frame of child and adolescent patients presenting with ASDs. In terms of clinical implications, although there is no current consensus regarding the treatment of children presenting with both ASDs and GV, this high rate of co-occurrence warrants more efforts in screening for GV among autistic patients, as well as autistic symptoms among those who present with GV. Furthermore, more focus should be placed on deciphering the appropriate and individualized classification of gender-related issues among children and adolescents diagnosed with ASDs. Facilitating our patients' experience and understanding of their gender identity and expression may be an important focus in treating clients with ASDs who also present with GV. It is hoped that future research may provide both scientific and clinical insights as to the nature of this phenomenon and best care strategies.

## Abbreviations Used

ASDs: autism spectrum disorders
CBCL: Child Behavior Checklist
GD: gender dysphoria
GID: Gender Identity Disorder
GV: gender variance
NOS: not otherwise specified
OR: odds ratio
